# Effects of temperature, salinity, and food availability on shell growth rates of the Yesso scallop

**DOI:** 10.7717/peerj.14886

**Published:** 2023-02-21

**Authors:** Alla V. Silina

**Affiliations:** National Scientific Center of Marine Biology, Far Eastern Branch of Russian Academy of Sciences, Vladivostok, Russia

**Keywords:** Mollusks, Bivalves, Scallops, Growth, Shell, Environment, Ecology

## Abstract

Shell growth rates in relationship to seasonal changes of environmental factors were studied in a wild population of the Yesso scallop *Mizuhopecten yessoensis* inhabiting Amur Bay (Peter the Great Bay, Sea of Japan, Russia). It was found that food availability is not a limiting factor for the scallop growth in the study area. A phytoplankton biomass of 3.5–6.0 g m^–3^ provided high scallop growth rates. The largest daily shell increments were observed with a phytoplankton biomass of about 6 g m^–3^. With a decrease in the phytoplankton biomass to <2 g m^–3^, as well as with an increase to >11 g m^–3^, the daily shell increments reduced. It appeared that the main exogenous factors causing the seasonal variations in the scallop growth rates are the water temperature, which was too high in July and August (>18 °C) and too low in November–April (<4 °C), and the water salinity, which was too low (<30‰) for this stenohaline species in summer. The relationship of the daily shell increment in Yesso scallop with the water temperature can be described by a dome-shaped curve. The largest increments were observed at 8–16 °C. The dependence of the daily shell increments on the water salinity was also best described by a dome-shaped curve, showing the optimal range of 32.5–33.5‰. The revealed relationships, approximated by dome-shaped curves, evidently indicate that both insufficient and excessive effect of the factor negatively affects scallop growth. A suggestion was made to describe the result of the combined impact of several environmental factors on the daily shell increment as a multiplication of the functions of its dependence on each of the factors.

## Introduction

An understanding of tolerance limits and optimal ranges of values of environmental factors for a species is important for addressing many theoretical and practical issues, including, in particular, its cultivation and conservation. The major environmental factors that affect growth of marine mollusks are water temperature and salinity, food composition and abundance, and oxygen concentration (MacDonald & Thompson, 1985; MacDonald & Thompson, 1986; Laing, 2000; Mao et al., 2022; Nan et al., 2022a). Tolerance limits and optimal environmental parameters for a species are usually determined in a laboratory, which has limitations regarding the reliability of the results obtained, as well as generally requiring labor and time-consuming methods. Therefore, in an aquarium, mollusks are usually kept under constant environmental conditions, a limited small set of food components is used, and so on (Duinker, Saout & Paulet, 2000; Martinez, Aguilera & Mettifogo, 2000). There is often non-flowing water in the aquarium, but it is known that the metabolism of mollusks is tightly coupled to fluctuations in ambient temperature (Pilditch & Grant, 1999). The growth rates are usually compared for two or three factor values. Depending on which factor values were chosen to compare the mollusk growth rates, different and sometimes opposite results can be obtained. Therefore, if the most studies reveal an increase in the growth rates with an increase in the water temperature, then there are also statements about the negative effect of increasing temperature on the scallop growth rates (Blicher, Rysgaard & Sejr, 2010).

Many studies of environmental influence on the scallop growth have been carried out under maricultural conditions (Nan et al., 2022a). The results of such studies have limitations for their generalization for the species. The main reason is the abnormally high population density of reared mollusks, which, in particular, leads to a change in the structure of the phytoplankton community due to a decrease in important food components in environment, which can lead to adverse consequences for the health and growth of mollusks (Yu et al., 2019). In addition, the oxygen concentration of the water may decrease, and the abundance of bacteria may increase, and so on (Newell, 2004).

When the object of research is in the wild, assessing the degree of effect of each factor separately becomes a challenge, as the organism is exposed to a multitude of factors that change throughout the year and, furthermore, frequently interdependent on each other. For example, a temperature increase leads to increased food consumption by animals in conditions of unlimited food supply. The solubility of oxygen in the water depends on the water temperature: the higher the latter, the lower the oxygen concentration in the water. It was shown that multiple environmental factors interact in unpredictable ways. The factors are more likely to act synergistically or antagonistically than additively (Crain, Kroeker & Halpern, 2008; Stevens & Gobler, 2018; Mao et al., 2022).

It would be valuable to study the links between mollusk growth rates and environmental parameters in the wild. It is possible to do this for bivalves because they form surface microgrowth rings and/or internal shell microgrowth patterns over certain time periods (Clark, 1974; Richardson, 2001). Knowledge of this period allows determine the growth rate per unit time and evaluate the relationship between growth rate and environmental factors controlling a shell deposition. However, the period of formation of microgrowths must be identified for each bivalve species. Some studies show daily rhythm of growth ring formation on the shell surface in the genera Pecten (Clark, 2005; Chauvaud, Thouzeau & Paulet, 1998; Chauvaud, 2005). To date, it has been established that from May to November, the Yesso scallop *Mizuhopecten yessoensis* deposits daily growth layers visible on the outer surface of its right valve (Silina, 1978).

Despite the pronounced seasonal fluctuations of environmental parameters in the habitat of such commercially valuable bivalve species as Yesso scallop, there is a scarcity of studies that compared scallop shell growth rates in different seasons and under different values of environmental parameters available in the literature. Previously, the dependence of shell growth rate on water temperature was studied. It was found that the growth rate increases with increasing temperature, but a too high water temperature leads to a negative effect, rather than to a stimulating one (Silina, 1983; Kosaka, 2016). The results of a study that considered the effects of food quantity as well as water salinity, on the growth of Yesso scallop as a stenohaline species have not been published. Although some studies have explored the influence of factors on Yesso scallop individually, the combined effects of two factors remain largely unknown.

The goals of the present work were as follows: (1) study the seasonal dynamics of shell growth rate in scallops from a wild population exposed to simultaneous changes in environmental parameters in order to identify the pattern of relationships of the scallop shell growth with the water temperature, water salinity, and food quantity, as well as determine the optimal ranges of these factors for the scallop shell growth; (2) investigate variations in the scallop growth rate under changes of two factors; (3) propose a model describing the dependence of the width of the daily increment on the environmental parameters under the combined impact of multiple factors on the scallop.

## Materials and Methods

**Figure 1:**
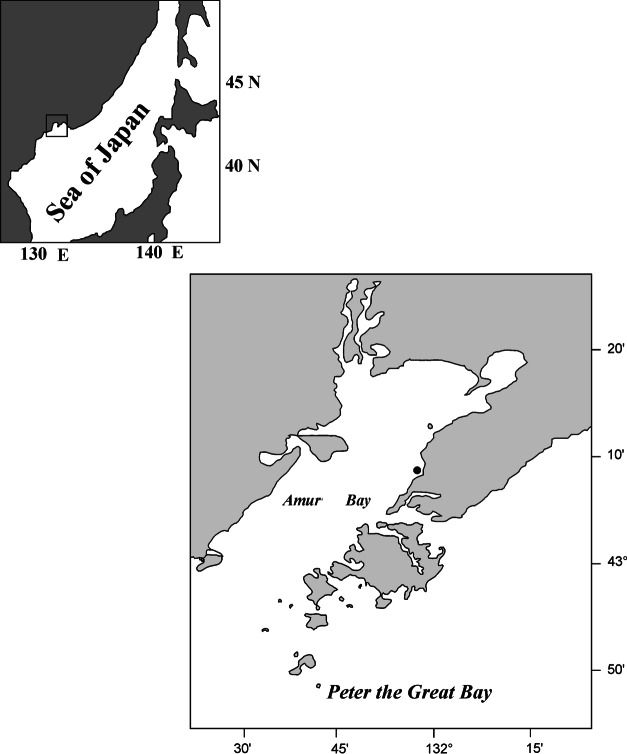
Maps of the scallop *Mizuhopecten yessoensis* sampling. The black circle indicates the site of scallop collection.

Yesso scallops were caught by divers at one site off the eastern coast of Amur Bay (Peter the Great Bay, Sea of Japan, East Sea) near the city of Vladivostok (43°09′N 131°56′E) from a depth of 6–8 m (Fig. 1). It is a monitoring station of the National Scientific Center of Marine Biology, Far Eastern Branch of Russian Academy of Sciences. The bottom sediments in the habitat of the wild scallop population consisted mainly of silted sand with pebbles. Scallops were collected in 1994, 1995, 1998 and 1999.

**Figure 2:**
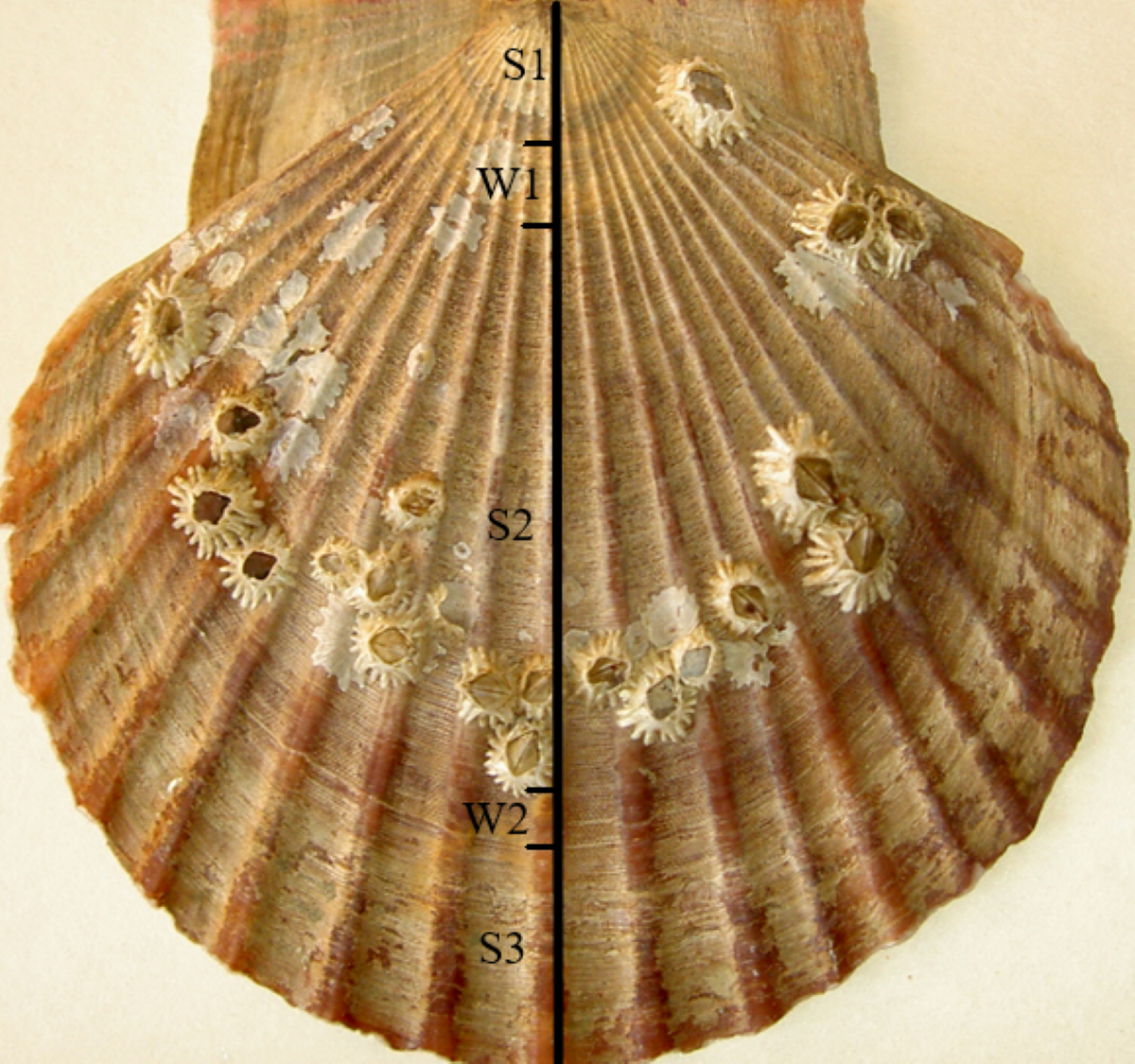
The outer surface of the upper (right) valve of two-year scallop *Mizuhopecten yessoensis*, C and W indicate the shell sections formed in the cold and warm seasons, respectively. The scallop was collected in October. The shell height is 98 mm.

**Figure 3:**
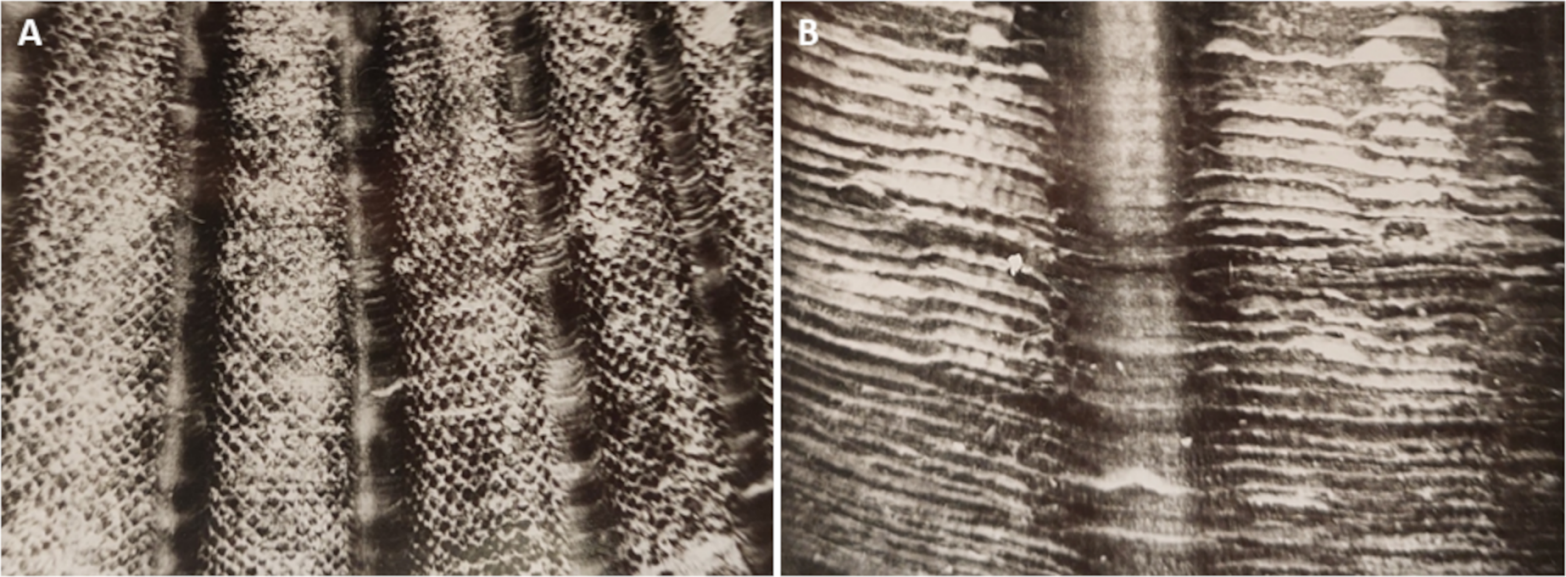
The microsculpture of the upper valve of the scallop *Mizuhopecten yessoensis* under binocular microscope. (A) shows the daily shell increments. (B) shows the weekly shell increments, the organic periostracum partially covers each of them.

The age of each scallop was determined by the microsculpture of the outer surface of the upper valve according to a method proposed earlier (Silina, 1978). This method is based on the results of the comparison of the appearance and width of elementary growth layers at the shell edges of the scallops collected in different months. Regular sampling of the scallops both from the wild and from cages revealed the seasonal periodicity of an alternation of areas with broad and narrow elementary growth layers on the outer surface of the right valve. In cold seasons (the water temperature <4 °C, from the end of November to the end of April), broad striped layers are formed, and in warm seasons (the water temperature >4 °C, the rest of the year), narrow layers of cellular appearance are built up (Figs. 2 and 3). By counting the number of concentric shell sections with elementary growth layers formed during the summer seasons, it is possible to determine the individual age of any scallop. There was correspondence between the number of narrow elementary growth layers and the number of the days in the warm seasons, evidence which is indicative of a daily deposition of each cellular elementary growth layer. The number of broad elementary growth layers gradually increased by four strips monthly during the cold seasons, that is, one strip was formed weekly. Knowing the durations of deposition of the layers, it is possible to determine the growth rate per unit time and to estimate the relationships between growth rate and environmental factors. Calculations and measurements of the widths of the concentric layers were performed along the valve central rib (Fig. 2) parallel to the growing shell edge under a binocular microscope using a measuring scale at a magnification of 20 ×. The boundary of the cellular layer is a line that can be drawn along the bulges far from the top of the shell. For cold seasons, the daily shell increase was calculated as the width of the weekly broad shell increment divided by seven. During a strong storm, the growing shell edge may break off a little, or the scallop closes tightly and stops growing. In this case, the number of distinguishable layers is reduced. In fast-growing juveniles, this decrease is usually 1–3 daily layers. The visible trace of such disruption of the growth remains on the valve surface for life.

It is known that somatic and reproductive growths are usually separated over time to maximize the reproductive output, so that the energy accumulated is primarily spent for sex products (MacDonald & Thompson, 1985; MacDonald & Thompson, 1986). Therefore, in order to exclude the predominant effect of the primary expenditure of resources for the growth of reproductive tissues, analysis of shell growth rate in Yesso scallop was conducted on individuals of the second year of life, since one-year-olds do not spawn, and the spawning of two-year-olds is low-productive in the study area (Silina, 2020). The dynamics of daily shell increments was calculated as the average value for five individuals that were at 1-yr of age in May 1993, as at the study site, Yesso scallops spawn once a year, in May (Dzyuba, 1986; Chung et al., 2005). Thus, the width of the daily scallop shell increment in each month was calculated as an average value for about 150 (5 ind. ×30 days) measurements of this indicator of the scallop growth rate.

In 1993, the abundance and biomass of phytoplankton were studied in the north-eastern part of Amur Bay at the monitoring station where the scallops were collected (Stonik & Orlova, 1998; Stonik & Orlova, 2002 (Station 9); Begun, Orlova & Zvyagintsev, 2003; Orlova, Stonik & Shevchenko, 2009; Aleksanin et al., 2012). Microalgae samples were taken twice a month during May–November 1993 (Stonik & Orlova, 1998). The study of the species composition of phytoplankton in Amur Bay revealed a total of 357 species of planktonic microalgae from eight divisions: Cyanophyta (eight species), Chrysophyta (eight), Bacillariophyta (157), Cryptophyta (five), Dinophyta (143), Raphidophyta (three), Euglenophyta (11), and Chlorophyta (22 species) (Orlova, Stonik & Shevchenko, 2009). While 116 algal genera were observed, usually the dominant genus represented about 60% of the sample’s biomass (minimum 20%) and four genera contributed about 90% of the total phytoplankton biomass. The fifteen leading genera in biomass included (in descending order of average biomass): *Thalassiosira, Coscinodiscus, Noctiluca, Pseudo-nitzschia, Chaetoceros, Thalassionema, Rhizosolenia, Eutreptia, Gymnodinium, Skeletonema, Prorocentrum, Ditylum, Diplopsalis, Dactyliosolen*, and *Protoperidinium* (Aleksanin et al., 2012). It is known that the diatoms constitute the major part of Yesso scallop’s diet (Silina & Zhukova, 2009). Microscopic analysis of plankton indicated the dominance of the diatoms *Thalassiosira* sp., *Rhizosolenia setigera*, *Pseudo-nitzschia* sp., *Chaetoceros* sp. and *Skeletonema costatum* (Stonik & Orlova, 1998). The highest density and biomass of phytoplankton were recorded in late July–early August. Such an abundance of phytoplankton was due to the development of diatoms *Skeletonema costatum.* The smallest biomass values were observed in autumn (Stonik & Orlova, 1998; Stonik & Orlova, 2002 (Station 9)). It is known that zooplankton is also an item of the scallop’s diet (Mikulich & Tsikhon-Lukanina, 1981; Silina & Zhukova, 2009). Yesso scallop is capable of catching particles in the size range from 9 to 950 µm (Mikulich & Tsikhon-Lukanina, 1981). The annual dynamics in the abundance of copepods and meroplankton were studied at the monitoring station in Amur Bay (Kulikova, Omel’yanenko & Pogodin, 1999; Chavtur & Kasyan, 2002). These data were used to assess the food supply of the habitat for the scallop during the year. The temperature of the near-bottom water was measured twice a month (three samples each) at the scallop collection site, and water samples were taken to evaluate its salinity in the laboratory. 1993 was an ordinary year in terms of environmental conditions, no extraordinary events occurred. An insignificant phytoplankton bloom had a short time span (about 2–3 days) and affected only the surface waters in the study area. To compare the scallop growth rate at different values of environmental factors, the width of the daily shell increment for each environmental indicator detected twice a month was calculated as an average value for approximately 75 (5 ind. ×15 days) measurements of the increments.

Statistical analysis was carried out in the Statistica 8.0 (software package). It included the method of principal components (factor analysis) to identify relationships and correlations between the factors and the daily shell increments. The curves that best represented the two-dimensional relationship of daily shell increments with each environment factor (curves factor *vs*. daily increments) and three-dimensional surfaces showing the relationship of daily shell increments with two factors (the quadratic surface factor 1 *vs*. factor 2 *vs*. daily increments) were selected by distance weighted least squares and negative exponential smoothing. These tests are suitable for the study and visualization of nonlinear relationships between scallop growth rates and environmental parameters. It is known that physiological processes are nonlinearly influenced by environmental conditions, therefore, substantially nonlinear relationships between the scallop shell proxies and the water temperature, as well as other factors are common, and they are difficult to describe in one mathematical equation (Bauwens et al., 2010).

## Results

### Seasonal dynamics of shell growth rate

**Figure 4:**
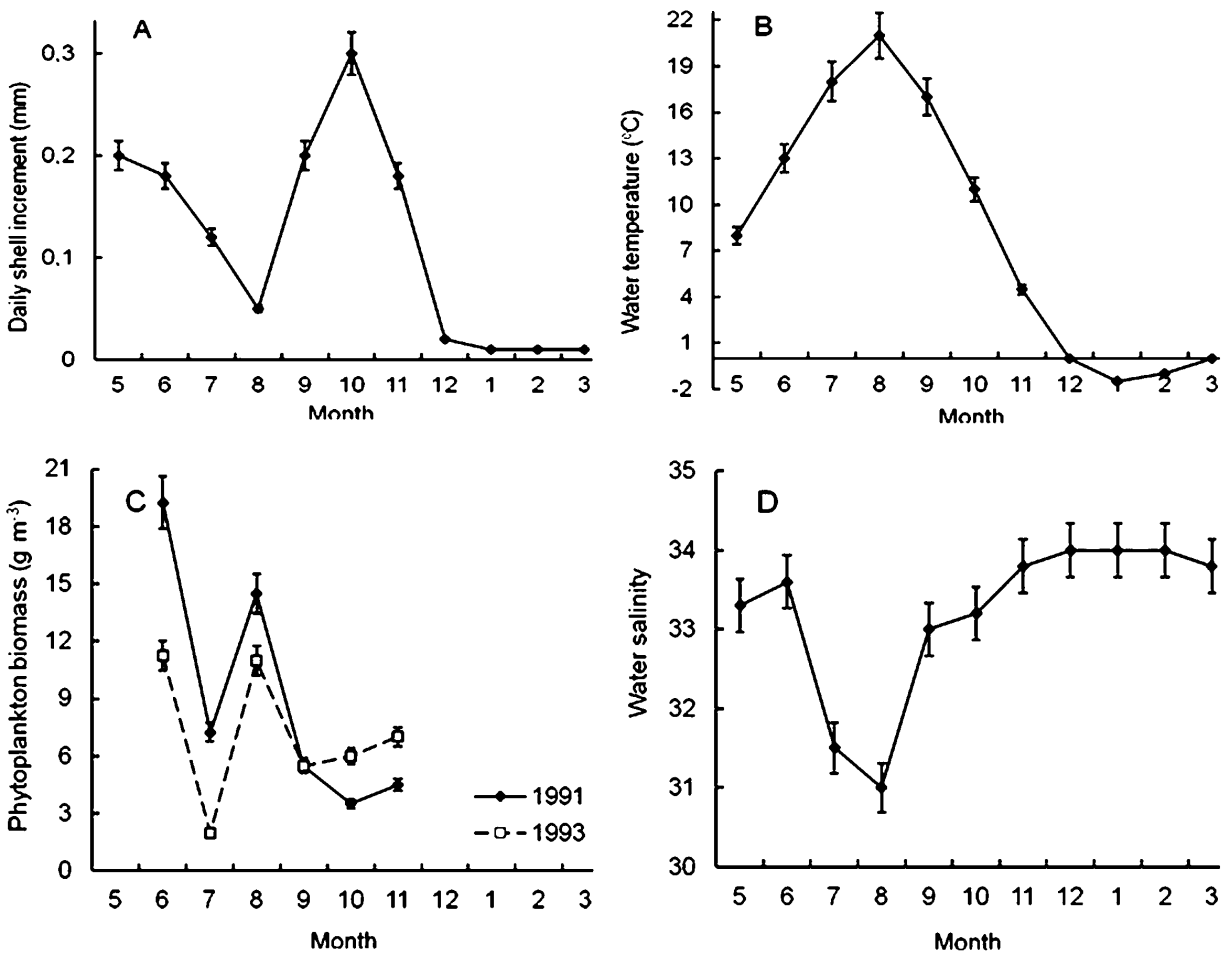
Seasonal dynamics of average values of the following environmental parameters at the scallop sampling site in the northeastern Amur Bay in 1993. (A) The daily shell increments of Yesso scallop *Mizuhopecten yessoensis* during the second year of life; (B) the temperature of the near-bottom water; (C) the phytoplankton biomass (according to Stonik & Orlova, 1998); and (D) the salinity of the near-bottom water. Vertical bars are errors of the average values of the parameters.

It was found that scallop shell grew unevenly during the year (Fig. 4A). The shell daily increments were quite high in May and June (Fig. 4A), when the water temperature and salinity varied in the range of 7–14 °C and 33–34‰, respectively (Figs. 4B, 4D). Later, in July and, especially, in August, the shell growth rates declined markedly (Fig. 4A). It is obvious that this decline was not due to a lack of food, since the phytoplankton biomass in the study area in August reached one of the highest values during the year (Fig. 4C). Apparently, there were at least two other factors unfavorable for Yesso scallop. First, the water temperature was above 18 °C (Fig. 4B). Second, these months were characterized by the lowest water salinity of the year, which also adversely affected the growth of the stenohaline Yesso scallop (Fig. 4D).

The shell growth rates significantly increased in autumn, but, obviously, this increase was not due to an increase in the food supply for the scallop, as there was a decrease in the abundance of phytoplankton in autumn, after the summer maximum of its abundance and biomass (Figs. 4A, 4C). The largest daily shell increments were recorded in October, when the water temperature was 8–16 °C, and the water salinity was stable and high (Figs. 4B, 4D). In October, the phytoplankton biomass decreased to 3.5–6.0 g m^−3^ (Fig. 4C).

The lowest average values of daily shell increments were observed at low water temperature, from late November to April (Figs. 4A, 4B). At the study site, phytoplankton always formed the second largest peak of its biomass in December–February (Aleksanin et al., 2012). The water salinity was stable and high, mainly in the range of 33–34‰(Fig. 4D). In winter, obviously, the low water temperature (<4°) was the limiting factor for the scallop shell growth.

### Relationship of the daily shell increments of Yesso scallop with each of the environmental factors

**Figure 5:**
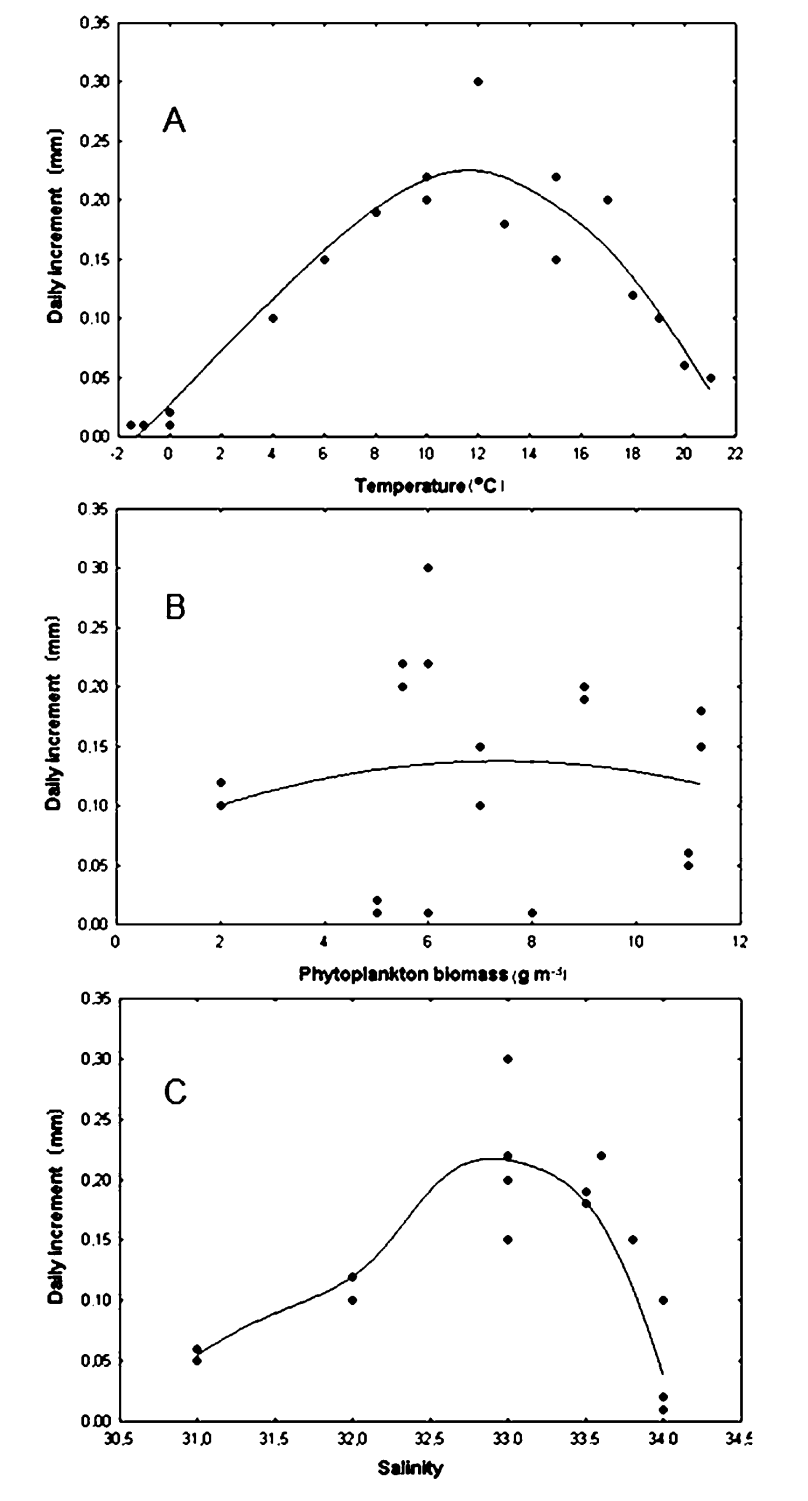
Relationship of daily shell increments of the scallop *Mizuhopecten yessoensis* with environmental factors. With (A) the temperature of the near-bottom water, (B) the biomass of phytoplankton (according to Stonik & Orlova, 1998) and (C) the salinity of the near bottom water at the scallop sampling site in Amur Bay in 1993. The solid lines are the curves that approximate the relationships.

The correlation coefficient between the daily shell increments of Yesso scallop and the water temperature was only +0.046 in the wide range of –2–23 °C, that is, this relationship was obviously nonlinear (Fig. 5A). Factor analysis approximated the relationship between the water temperature and the daily shell increments as a dome-shaped curve (Fig. 5A). The approximation curve was revealed as the average distance between points using distance weighted least squares, according to the statistical test. The scatter of points was determined by the influence of other environmental factors on growth. In this regard, another curve was of interest: the curve that was located directly above (to envelop the top of) all points, close to the topmost points of the relationship. It would most realistically describe the pattern of dependence of the daily shell increments on the water temperature. It could only be achieved with optimal values of other factors. Below this curve, points were located that correspond to daily shell increments under the effect of other factors that did not have optimal values for the species, therefore slowing the scallop’s growth down. Based on the results obtained, such curve was also visually represented by a dome-shaped curve (Fig. 5A). The maximum value of daily shell increments was observed at a temperature located practically in the middle of the variation range (Fig. 5A). A significant decrease in shell growth rates was clearly expressed both when the water temperature dropped below 4 °C and when it increased above 18 °C (Fig. 5A). Thus, in the study area, the water temperature can limit scallop growth not only in the winter–early spring period, when it is low, but also in July and August, when it reaches the highest values (Fig. 5B).

It was found that the daily shell increment was non-linearly dependent on phytoplankton biomass (r = +0.028) for the wide range of food abundance in the study area (Fig. 5B). This relationship was approximated by a wide dome-shaped curve (Fig. 5B). The result showed that the curve, which was located close to the uppermost points, that is, realistically described the dependence of the daily shell increments only on phytoplankton biomass, and was also visually represented by a dome-shaped curve (Fig. 5B). The largest daily shell increments were observed with a phytoplankton biomass of about 6 g m^−3^. With a decrease to <2 g m^−3^, as well as with an increase to >11 g m^−3^, the shell growth rates decreased (Fig. 5B).

The correlation coefficient between the daily shell increments and the bottom water salinity in the range of 31–34‰ was low (r = +0.021). The growth response of the scallop to variations in this factor was nonlinear (Fig. 5C). Factor analysis also approximated this relationship by a dome-shaped curve, showing the optimal range of 32.5–33.5‰ for the studied stenohaline bivalve species (Fig. 5C). The results showed that the curve, which was located close to the topmost points, was also visually represented by a dome-shaped curve (Fig. 5C). A significant reduction in shell growth rates was observed when the salinity decreased below 32.0‰ (Fig. 5C). Low values of daily shell increments at a high water salinity, 34.0‰ (Fig. 5C), were most likely due to the low winter water temperature for the species, since the stable water salinity of approximately 34.0‰ in the study area is usually observed during the cold season (Figs. 4B, 4D). This is the result of the combined simultaneous effect of two factors on scallops in the natural environment.

### Multiplicative effect of two factors on daily shell increments in Yesso scallop

**Figure 6:**
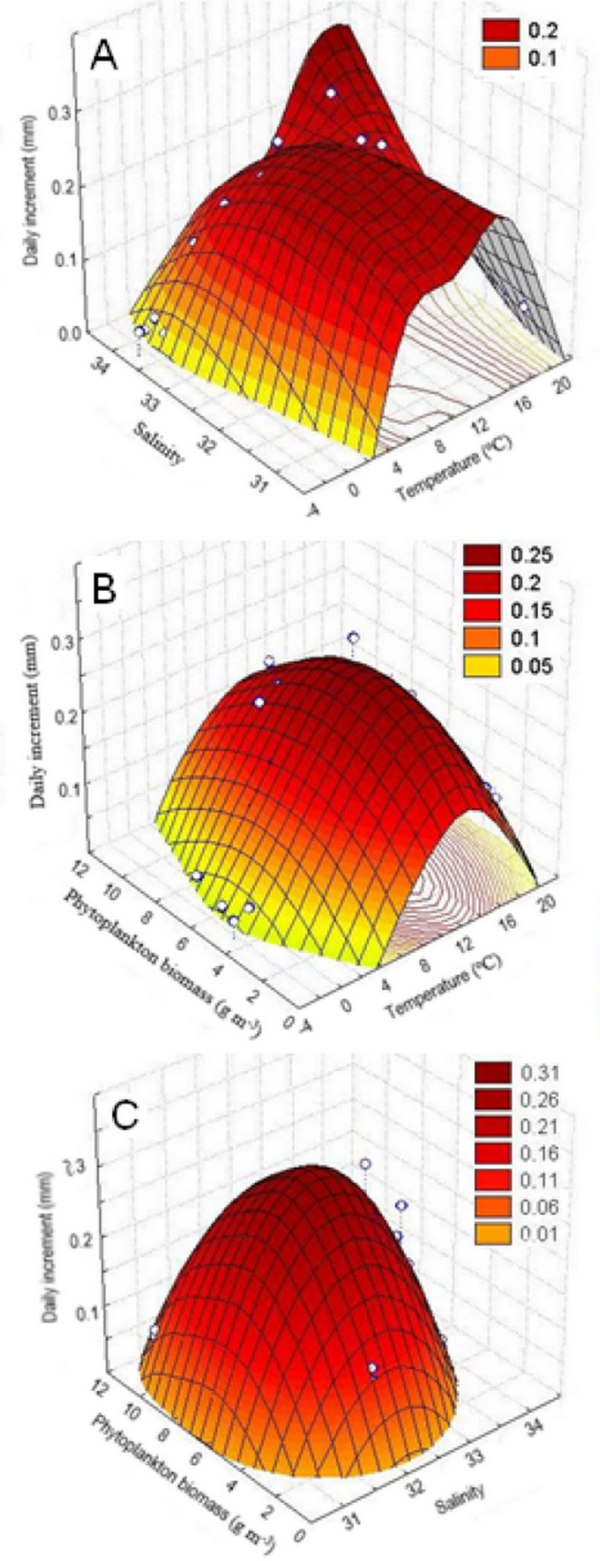
Three-dimensional relationship of daily shell increments of the scallop *Mizuhopecten yessoensis* in the second year of life with the following environmental parameters at the scallop sampling site in Amur Bay in 1993. (A) The salinity and temperature of the near-bottom water; (B) the temperature of the near-bottom water; and (C) phytoplankton biomass (according to Stonik & Orlova, 1998), and the phytoplankton biomass and the near-bottom water salinity.

The study of the combined effect of various environmental factors on Yesso scallop growth by the method of principal components (factor analysis), the quadratic surface of temperature *vs*. salinity *vs*. daily increments also demonstrated a dome-shaped relationship of the shell growth rates with both temperature and salinity of water. The largest daily shell increments were in the ranges of 6–18 °C and 32.5–34.0‰, respectively, for the water temperature and salinity (Fig. 6A). In addition, the range of water temperature favorable for growth provided to be narrower with the deterioration of salinity conditions in the habitat (Fig. 6A). Thus, it was revealed that these factors act synergistically.

The quadratic surface of temperature *vs*. phytoplankton biomass *vs*. daily increments confirmed dome-shaped relationship of the shell growth rates with the water temperature, as well as with the phytoplankton biomass, and showed a narrower range of water temperatures favorable for scallop shell growth at low and high phytoplankton abundances (Fig. 6B). Thus, it was revealed that these factors act synergistically, too. Based on the concentric ellipsoid curves on the projection of the daily increments’ surface onto the temperature *vs*. phytoplankton biomass plane, the largest increases were observed in the ranges of 8–16 °C and 4–8 g m^−3^, respectively (Fig. 6B).

The quadratic surface of salinity *vs*. phytoplankton biomass *vs*. daily increments showed a decrease in shell growth rate, both with a decrease and increase in values of these factors close to the limit values observed for the study site (Fig. 6C). The range of such factor as phytoplankton biomass was determined as most favorable for scallop shell growth (4–10 g m^−3^) by the projection of the surface onto the salinity *vs*. phytoplankton biomass plane. As regards water salinity, the optimal range was revealed as 32.0–33.0‰ (Fig. 6C). The following relationships were revealed: (1) a sharp reduction in shell growth rates was recorded in the case of a low phytoplankton biomass, and (2) the scallop shell growth was possible only in a narrow range of water salinity (Fig. 6C).

## Discussion

The growth rates of Yesso scallop shell has been found to change significantly during the year, in accordance with the seasonal variations in the environmental parameters. However, not all factors are of equal ecological importance for scallop. It is known that the growth rate characteristic of an animal species depend primarily on the amount of food consumed, which is determined by the concentration and quality of food available for this species in the habitat (MacDonald & Thompson, 1985). It has been found that the main period of scallop shell growth is from late September to mid-November. It is timed to the season of the most rapid growth of somatic soft tissues in this bivalve (Silina, 2020). However, this period of rapid growth does not coincide in time with the season of maximum phytoplankton biomass, including that of diatoms (the main food item in the diet of Yesso scallop (Silina & Zhukova, 2009) in the study area (Stonik & Orlova, 1998; Stonik & Orlova, 2002; Aleksanin et al., 2012). As for zooplankton, another major item in the scallop’s diet (Mikulich & Tsikhon-Lukanina, 1981; Silina & Zhukova, 2009), the biomass of meroplankton and copepods in Amur Bay does also not reach maximal values in autumn either (Kulikova, Omel’yanenko & Pogodin, 1999; Chavtur & Kasyan, 2002). Moreover, it is known that the density of the non-diatomic component of phytoplankton (dinoflagellates and euglenides) had increased in Amur Bay in the 1990s, especially in spring and autumn (Stonik & Orlova, 1998; Stonik & Orlova, 2002), which also reduces the quality of food in these seasons, because non-diatoms constitute a minor part of Yesso scallop diet (Silina & Zhukova, 2009). At the same time, it has been found that in summer, when the maximum phytoplankton biomass is recorded, mainly due to diatoms (Stonik & Orlova, 1998; Stonik & Orlova, 2002), values of shell growth rates are low. The weight of somatic soft tissues in Yesso scallop also decreases in summer (Silina, 2020). Meroplankton is abundant in April and August, not in October (Kulikova, Omel’yanenko & Pogodin, 1999). Thus, the quantity and quality of food in this area is not a limiting factor for the scallop growth almost throughout the year.

In this work, it was revealed for the first time that 3.5–6.0 g m^−3^ of phytoplankton biomass provides a high scallop growth rates. The scallop growth rates decrease when the phytoplankton biomass in the habitat decreases or exceeds this range. The significant decrease in scallop growth rates with a phytoplankton biomass <2.0 g m^−3^ can be explained by insufficient food availability for the species. However, the considerable decrease in growth rates with a phytoplankton biomass >11.0 g m^−3^ is most likely due to the fact that a high concentration of small particles in the water can lead to “blocking” (clogging) of gills in bivalves, thus, preventing normal filtration and, consequently, feeding and respiration (Chauvaud, Thouzeau & Paulet, 1998), which inevitably affects their growth. Besides, large amount of dead plankton induces the proliferation of bacteria that mineralize organic matter. Microbial respiration reduces the oxygen content of the near-bottom water and sediments (Newell, 2004). In addition, the concentration of oxygen in the water decreases in the dark due to the consumption of oxygen by phytoplankton, which also negatively affects the physiological condition of Yesso scallop, since this mollusk is sensitive to reduced oxygen concentration in the water, the oxygen concentration in the water of <6 ml l^−1^ is insufficient for this bivalve (Yamamoto, 1957). Similar results were obtained for the scallop *Chlamys farreri*. It stopped growing when particular organic matter (POM) was <0.90 mg l^−1^ and grew rapidly with increasing POM, however, when POM was >3.67 mg l^−1^, the growth rates of this scallop decreased again (Yang et al., 1999). Lodeiros with co-authors (2012) carried out growth trials on the scallop *Euvola ziczac* during two short-term (34–36 days) periods and showed that its growth was enhanced at abundant phytoplankton biomass. It is possible that these two biomass values were chosen from the left (ascending) half of the real dependence of the scallop growth rates on the abundance of food in the environment.

For a poikilothermic animal, one of the most important environmental factors is temperature. Laing (2000) argued that temperature rather than food is the factor most often limiting for growth in the sea. Indeed, one of the major factors that caused seasonal changes in the shell growth rates of Yesso scallop in Amur Bay was the water temperature: it was too high for the species in July–August (>18 °C) and too low in November–April (<4 °C).

In this work, it is found that the tolerable water temperature range for Yesso scallop is from –2 to 23 °C, and the largest daily shell increments are formed at 8–16 °C. Kosaka (2016) showed that the tolerant temperature for Yesso scallop is 4–23 °C, while the optimal temperature ranges from 10 to 15 °C in warm waters off the coasts of Japan. Observations on the growth of 1–3-year-old scallops cultivated in the warm waters off Honshu Island (Japan) also showed a slowdown of the shell growth in July–September at a water temperature of >20° (Fuji & Hashizume, 1974). Using a cardiac performance monitoring system, Xing et al. (2016) demonstrated that thermal limit of Yesso scallop is about 22 °C, which is consistent with my findings.

The study on Yesso scallop growth in wild in different seasons within appropriate natural ranges of the water temperatures revealed that the relationship between the water temperature and the daily shell increments has a dome-shaped form. A similar result was obtained for the scallop *Argopecten ventricosus circularis*, as well as for the oysters *Pinctada margaritifera* and *P. maxima* (Sicard et al., 1999; Yukihira, Lucas & Klumpp, 2000). It follows that if in the experiment the temperature values are chosen in the ascending part of the real dependence of the scallop growth rates on the water temperature, then a conclusion will be made about a positive and possibly even linear relationship between growth and temperature. This is the most common case. If one temperature value is selected in the ascending part, and the other value is located in the descending part of the real dependence of the scallop growth rates on the water temperature, then it can be concluded that the growth rate is independent of temperature. Indeed, sometimes the independence of growth from changes in the water temperature is revealed. So, Navarro with co-authors (2000) suggested that a scope for growth in *Argopecten. purpuratus* does not depend on temperature. If the temperature values are selected from the descending part of the real dependence of the scallop growth rates on the water temperature, then a conclusion can be made about the negative dependence of the growth rates on temperature. For instance, Blicher with co-authors (2010) examined seasonal variation in the growth rates of the scallop *Chlamys islandica* and revealed that elevated temperature reduces growth.

Since Yesso scallop is a stenohaline animal, the result about the great importance of water salinity for its growth was quite expected. It was found that scallop growth slows down to a high degree when the salinity decreased below 32.0‰. Salinity and other factors act synergistically on the scallop growth, producing outcomes that could not be predicted from the responses to individual factors. Earlier, it was also suggested a synergistic effect of two stressors on some bivalve species (Crain, Kroeker & Halpern, 2008; Stevens & Gobler, 2018; Mao et al., 2022).

The revealed relationships of daily shell increments with each of the three environmental factors important for the poikilothermic stenohaline Yesso scallop, which are approximated by dome-shaped curves, clearly indicate that both insufficient and excessive effect of the factor negatively affects scallop growth. In wild populations, even under optimal values of one or more factors, scallop daily shell increments can be extremely low. Thus, in December–February, Yesso scallop has enough food available to maintain its high growth rates, with the water salinity being favorable for the species; nevertheless, its daily shell increments are small due to the low water temperature. Moreover, the results of the study showed a narrowing of the water temperature range that favorable for the scallop growth both when the salinity conditions deteriorate, and when the phytoplankton abundance is too low or high for this bivalve. These are the examples of a combined simultaneous effect of various factors on an organism in nature. According to the Leibig’s law of the minimum, the Blekhman’s law of limiting factors and the Shelford’s law of tolerance (the law of optimum), the success of an organism depends on a complex of conditions in the habitat, while the inability to thrive is determined by an insufficiency or, conversely, an excess of any factor (Shelford, 1913; Odum, 1983). Therefore, based on the results obtained and on these well-known laws, I propose to describe mathematically the result of the multiplicative (combined) effect of several environmental factors on the daily shell increment as a product of the functions of its dependence on each of the factors:

\begin{eqnarray*}{I}_{\mathrm{t}}=a\times F({x}_{1})\times F({x}_{2})\times F({x}_{3})\times ..., \end{eqnarray*}

where I_t_ is the increment for a t-th day. Constant *a* is the maximum possible daily shell increment for the species in the study area. For the population under study, *a* = 0.4 mm. F(x_i_) is the function of the dependence of the daily shell increment on the i-th factor; x_i_ is the value of the i-th factor in t-th day.

For such description, it is necessary to select the most important factors for this species in the study area, as not all factors are of equal ecological significance. Based on the results of the study, three factors have been selected to describe the growth of the poikilothermic stenohaline Yesso scallop: the food availability, the water temperature, and the water salinity. Some additional factors may also become particularly important in some areas, such as oxygen concentration in zones with high organic matter content in the water caused by anthropogenic pollution or under mariculture (Yang et al., 2021). In the conditions of Yesso scallop mariculture, even some unusual factors may become important, for example, the regional average air temperature in early winter (Nan et al., 2022b).

To successfully use the proposed model, the following conditions should be satisfied for the selection of functions: (1) 0 ≤ F(x_i_) ≤ 1; (2) F(x_iopt_) = 1, or close to 1 at the optimum value of the i-th factor (x_iopt_); (3) F(x_i_) should tend to 0 when approaching the lower or upper limit of the i-th factor in the tolerance range for the species; (4) the types of functions are determined from empirical observations. When these conditions for selecting the type of functions are met, the growth rates will be maximum (equal or close to the constant *a*) only under the full set of favorable factors. If there is a lack or excess of at least one of the factors, the growth rate will slow down, this is consistent with the revealed fact that the factors act synergistically. In case of two factors, the regions of acceptable values of the factors that provide a certain level of growth rates will look close to ellipses. Studies of the multiplicative effects of two factors on the shell growth rates of Yesso scallop showed that the projections of the quadratic surfaces on the factors’ planes really look like ellipses (Figs. 6B, 6C).

##  Supplemental Information

10.7717/peerj.14886/supp-1 Supplemental Information 1 Measurements of the daily shell increments of Yesso scallop, and the results of measurements of the water temperature and salinity

## Additional Information and Declarations

### Data Availability

The following information was supplied regarding data availability: The raw measurements are available in the Supplementary File.
